# University Publishing Company—New York and Baltimore

**Published:** 1875-01

**Authors:** 


*University Publishing Company—New York and Baltimore.*

We call attention to the University Series of school books advertised on the second page of the cover of this journal. It will be be a source of pride to every patriot when not every section of this country, but every State, can furnish its own literature and school publications. Our brethren of other sections of our Union should be as justly proud of our achievements in this direction as we are of theirs. We hope our readers will present the claims of the books published at this house. We will have more to say on this subject.

